# 异基因造血干细胞移植治疗510例活动性复发难治性急性髓系白血病的真实世界研究

**DOI:** 10.3760/cma.j.cn121090-20260120-00040

**Published:** 2026-06

**Authors:** 孝永 满, 明璐 李, 新红 费, 昊钰 程, 宇明 殷, 荣牡 罗, 书芹 张, 江英 顾, 松 薛, 维婕 张, 帆 杨, 其航 满, 怡欣 杨, 正芹 田, 婷婷 李, 磊 袁, 红霞 温, 薇 危, 文静 汪, 岩 王, 静波 王

**Affiliations:** 1 航天中心医院血液科，北京 100049 Department of Hematology, Aerospace Center Hospital, Beijing 100049, China; 2 北京化工大学校医院，北京 100029 Department of Bone Marrow Transplantation, Beijing Lu Daopei Hospital, Beijing 101102, China; 3 北京陆道培医院，北京 101102 University Hospital, Beijing University of Chemical Technology, Beijing 100029, China

**Keywords:** 白血病，髓系，急性, 复发难治性, 活动性, 异基因造血干细胞移植, Leukemia, myeloid, acute, Relapsed/refractory, Active disease, Allogeneic hematopoietic stem cell transplantation

## Abstract

**目的:**

探讨活动性复发难治性急性髓系白血病（R/R AML）患者接受挽救性异基因造血干细胞移植（allo-HSCT）的长期生存结局及其影响因素。

**方法:**

本研究为回顾性真实世界研究。选取2012年7月至2024年12月于航天中心医院接受allo-HSCT的510例R/R AML患者，所有患者移植前骨髓原始细胞比例均≥5％。采用Kaplan-Meier法评估总生存（OS）和无病生存（DFS），采用竞争风险模型分析累积复发率（CIR）及非复发死亡率（NRM）。通过单因素Log-rank检验及多因素Cox回归模型和Fine-Gray模型筛选影响预后的独立危险因素。

**结果:**

中位随访时间为50（0～151）个月，510例患者预估10年OS率和DFS率分别为35.5％（95％*CI*：31.2％～40.5％）和33.2％（95％*CI*：29.1％～38.0％）。多因素分析显示，ELN风险分层是影响预后的核心因素，与低危组相比，中危及高危患者OS和DFS均显著降低（OS：*HR*＝1.74、1.97，*P*＝0.002、<0.001；DFS：*HR*＝1.77、1.99，均*P*<0.001）。发病至移植时间≤10个月与更好的OS和DFS独立相关（OS：*HR*＝0.64，*P*<0.001；DFS：*HR*＝0.62，*P*<0.001），而CD34⁺细胞输注量不足（≤4.3×10⁶/kg）是OS和DFS的不良预后因素（OS：*HR*＝1.57，*P*<0.001；DFS：*HR*＝1.62，*P*<0.001）。在竞争风险分析中，ELN高危分层（*HR*＝2.90，*P*<0.001）、移植前骨髓原始细胞比例≥50％（*HR*＝1.62，*P*＝0.037）以及CD34⁺细胞输注量不足（*HR*＝1.64，*P*＝0.003）与较高的累积复发率（CIR）独立相关；Ⅲ～Ⅳ级急性GVHD（*HR*＝0.54，*P*＝0.033）及轻、中、重度cGVHD（*HR*分别为0.38、0.13、0.24，*P*分别为<0.001、<0.001、0.015）均与较低CIR相关。对于非复发死亡，Ⅲ～Ⅳ级aGVHD（*HR*＝2.36，*P*<0.001）是主要危险因素，年龄≤35岁患者非复发死亡风险较低（*HR*＝0.68，*P*＝0.033）。

**结论:**

对于移植前仍存在活动性的R/R AML患者，直接行挽救性allo-HSCT仍可能获得长期无病生存。长期预后主要由疾病生物学特征及移植后免疫效应共同决定，其中ELN风险分层和GVHD在结局中起主导作用，而移植前肿瘤负荷主要影响复发风险但并非长期生存的独立决定因素。因此在临床实践中应更加重视风险分层及移植时机选择，而非单纯追求移植前完全缓解。

## Abstract (en)

**Objective:**

To investigate the long-term survival outcomes of salvage allogeneic hematopoietic stem cell transplantation（R/R AML）.

**Methods:**

This was a retrospective real-world study. We conducted a retrospective analysis of 510 patients with R/R AML who underwent allo-HSCT at Aerospace Center Hospital between July 2012 and December 2024. All patients had active disease before transplantation, defined as a bone marrow blast proportion of at least 5％. Overall survival（OS）and disease-free survival（DFS）were assessed using the Kaplan-Meier method, whereas the cumulative incidence of relapse（CIR）and non-relapse mortality（NRM）were evaluated by competing-risk models. Univariate analysis was performed using the log-rank test, and multivariable analysis was conducted using Cox regression for survival outcomes and Fine-Gray regression for competing-risk outcomes to identify independent prognostic factors.

**Results:**

In this cohort of 510 patients, the median follow-up duration was 50 months（range, 0–151 months）. The estimated 10-year overall survival（OS）and disease-free survival（DFS）rates were 35.5％（95％ *CI*: 31.2％–40.5％）and 33.2％（95％ *CI*: 29.1％–38.0％）, respectively. Multivariable analysis demonstrated that European LeukemiaNet（ELN）risk stratification was a core prognostic factor. Patients in the intermediate-risk（*HR*＝1.74, *P*＝0.002）and adverse-risk（*HR*＝1.97, *P*<0.001）categories had markedly lower OS and DFS rates. Mild-to-moderate chronic graft-versus-host disease（cGVHD）served as a protective factor for OS and DFS（both *P*<0.001）. A diagnosis-to-transplant interval longer than 10 months（*HR*＝1.56, *P*<0.001）was correlated with poor survival outcomes. A higher infused CD34^+^ cell dose（≥4.3×10⁶/kg）was independently associated with superior OS（*HR*＝0.64, *P*<0.001）and DFS（*HR*＝0.62, *P*<0.001）. In competing-risk analysis, intermediate and adverse ELN risk stratification（*HR*＝2.73 and 2.90, respectively; both *P*<0.001）and elevated bone marrow blast percentage（≥50％）（*HR*＝1.62, *P*＝0.037）were independently linked to increased relapse risk. Mild, moderate, and severe cGVHD, compared with no cGVHD（*HR*＝0.38, 0.13, and 0.24; *P*<0.001, *P*<0.001, and *P*＝0.015, respectively）, and grade Ⅲ–Ⅳ acute graft-versus-host disease（aGVHD）（*HR*＝0.54, *P*＝0.033）were associated with a lower CIR. Regarding non-relapse mortality（NRM）, grade Ⅲ–Ⅳ aGVHD（*HR*＝2.36, *P*<0.001）and severe cGVHD（*HR*＝2.74, *P*<0.001）were major risk factors, and advanced age（*HR*＝1.47, *P*＝0.033）was also associated with higher NRM.

**Conclusion:**

This study indicates that long-term disease-free survival may be attained through salvage allo-HSCT in patients with R/R AML despite active disease at transplantation. Long-term outcomes are mainly determined by disease biology and post-transplant immune effects, with ELN risk stratification and GVHD playing dominant roles, whereas pre-transplant tumor burden mainly affects relapse risk but is not an independent determinant of long-term survival. These findings suggest that greater emphasis should be placed on risk stratification and transplantation timing in clinical practice, rather than solely pursuing complete remission prior to transplantation.

随着免疫靶向药物的应用，急性髓系白血病（AML）的完全缓解（CR）率显著升高[1]–[4]，缓解期行异基因造血干细胞移植（allo-HSCT）已被证实可为患者带来明确的生存获益。但是经过各种化疗方案及靶向治疗未缓解（NR）的复发/难治性AML（R/R AML）患者是否应该直接进行allo-HSCT目前仍存在争议[5]–[7]。基于这一临床需求，本中心开展了一项为期12年的回顾性真实世界研究，重点分析移植前NR状态的患者行挽救性移植的疗效和临床可行性，旨在进一步优化移植治疗体系，为NR状态患者移植决策提供参考依据。

## 对象与方法

一、研究对象

本研究为回顾性真实世界研究，分析2012年7月至2024年12月于航天中心医院血液科接受allo-HSCT的R/R AML患者510例，其中接受单倍体移植382例，同胞全合移植81例、非血缘移植33例、脐血移植14例。入组标准：R/R AML患者、移植前骨髓原始细胞均≥5％，R/R AML的诊断符合《中国复发难治性急性髓系白血病诊疗指南（2023年版）》标准[8]。除外急性混合细胞白血病、AML-M_7_、骨髓增生异常肿瘤（MDS）、慢性髓细胞性白血病加速期患者。依据2022年欧洲白血病网（ELN）制定的AML预后分型标准，对研究对象进行预后风险分层[9]。患者临床特征详见表1。本研究经航天中心医院伦理委员会审查批准（批件号：20200511-JJJHZ-02）。

**表1:** 接受异基因造血干细胞移植治疗的510例活动性复发难治性急性髓系白血病患者基本临床资料

临床特征	数值
移植时年龄［岁，*M*（*IQR*）］	35.0（24.0, 48.0）
供者年龄［岁，*M*（*IQR*）］	35.0（24.0, 44.5）
发病至移植间隔［月，*M*（*IQR*）］	10（6, 16）
MNC输注量［×10⁸/kg，*M*（*IQR*）］	9.09（8.55, 9.79）
CD34^+^细胞输注量［×10^6^/kg，*M*（*IQR*）］	4.30（2.90, 6.70）
性别［例（％）］	
男	283（55.5）
女	227（44.5）
ELN风险分层［例（％）］	
低危	103（20.2）
中危	203（39.8）
高危	204（40.0）
M_5_亚型［例（％）］	
是	143（28.0）
否	367（72.0）
伴中枢神经系统白血病［例（％）］	
是	39（7.6）
否	471（92.4）
骨髓原始细胞比例［例（％）］	
5％～<20％	115（22.5）
20％～<50％	149（29.2）
≥50％	246（48.2）
移植类型［例（％）］	
单倍体	382（74.9）
同胞全合	81（15.9）
非血缘	33（6.5）
脐血	14（2.7）
移植物来源［例（％）］	
BM+PB	264（51.8）
PB	232（45.5）
UCB	14（2.7）

**注** MNC：单个核细胞；ELN：欧洲白血病网；BM：骨髓；PB：外周血；UCB：脐血

二、治疗方案

1. 预处理方案：CLAG（克拉屈滨+阿糖胞苷+G-CSF）+白消安+环磷酰胺方案178例，全身放射治疗（TBI）+CLAG方案114例，TBI+FLAG（氟达拉滨+阿糖胞苷+G-CSF）方案49例，TBI+环磷酰胺方案44例，白消安+环磷酰胺方案99例，后置环磷酰胺方案26例。

2. 移植物抗宿主病（GVHD）的预防和治疗：所有患者均采用短程甲氨蝶呤、环孢素、吗替麦考酚酯为基础的GVHD预防方案。针对GVHD的治疗包括一线治疗：糖皮质激素联合钙调磷酸酶抑制剂；二线标准治疗：糖皮质激素联合巴利昔单抗。

三、定义及标准

植入标准：中性粒细胞连续3 d≥0.5×10^9^/L，血小板脱离输注后连续7 d≥20×10^9^/L。急性GVHD（aGVHD）按照改良Glucksberg标准[10]进行诊断和分级。慢性GVHD（cGVHD）根据2014年美国国立卫生研究院共识标准[11]进行诊断和分级。活动性疾病即移植前骨髓原始细胞≥5％。总生存（OS）时间定义为造血干细胞回输当日至死亡时间或末次随访时间。无病生存（DFS）时间定义为造血干细胞回输当日至骨髓复发时间或死亡时间。复发则定义为获得CR后骨髓再次出现原始细胞≥5％或出现病理学证实的髓外病变。非复发死亡率（NRM）定义为非原发疾病复发或疾病进展导致的死亡发生率。

四、随访

随访截止日期为2025年5月1日。通过查阅患者住院病历、门诊随访记录和电话随访获得患者生存资料。

五、统计学处理

统计学分析采用*R*软件（版本4.4.2）完成。双侧*P*<0.05认为差异具有统计学意义。连续变量以中位数（*IQR*）表示，分类变量以例数（百分比）表示。对存在缺失的协变量，采用多重插补方法进行处理，基于预测均值匹配（predictive mean matching, PMM）生成5个插补数据集（*m*＝5），迭代10次后收敛。所有回归分析结果基于各插补数据集分别建模后，采用Rubin法则进行合并。OS和DFS采用Kaplan-Meier法进行估计，组间差异通过Log-rank检验进行比较；Cox比例风险回归模型用于单因素及多因素分析。累积复发率（CIR）和NRM采用累积发生函数（cumulative incidence function, CIF）进行估计，其中复发与非复发死亡互为竞争事件；组间差异采用Gray检验进行比较；多因素分析采用Fine-Gray比例亚分布风险模型进行评估。aGVHD、cGVHD及巨细胞病毒（CMV）血症的发生率亦采用CIF方法估计，死亡作为竞争风险事件，组间比较采用Gray检验。多因素分析中纳入的变量包括：单因素分析中*P*<0.10的变量以及临床上已知与预后相关的重要因素。对于存在嵌套关系或多重共线性的变量（方差膨胀因子>5），仅保留其中一个变量纳入模型分析。

## 结果

1. 造血重建及相关并发症：除12例患者因早期死亡未发生中性粒细胞植入，余均获得中性粒细胞植入，植入中位时间14（8～37）d。100 d内共425例（83.3％）患者获得血小板植入，植入中位时间15（10～93）d，85例血小板未植入；100 d血小板累积植入率86.3％（95％*CI*：83.4％～89.2％）。aGVHD累积发生率45.1％（95％*CI*：40.8％～49.4％），中位发生时间为36（7～100）d；其中Ⅱ～Ⅳ级aGVHD累积发生率31.6％（95％*CI*：27.5％～35.6％）；Ⅲ～Ⅳ级aGVHD累积发生率13.7％（95％*CI*：10.7％～16.7％）。随访期间共有204例发生cGVHD，其中轻度111例，中度76例，重度17例；cGVHD的累积发生率为41.8％（95％*CI*：37.4％～46.1％）。264例患者发生CMV血症，其中100 d累积发生率为51.8％（95％*CI*：47.4％～56.1％）。

2. 复发及生存：共有7例（1.37％）移植后NR，此外162例（31.7％）移植后复发，复发中位时间为4（1～39）个月，预估10年CIR为34.9％（95％*CI*：30.6％～39.2％）。移植后预估10年OS率为35.5％（95％*CI*：31.2％～40.5％）；10年DFS率为33.2％（95％*CI*：29.1％～38.0％）（图1）。

**图1:**
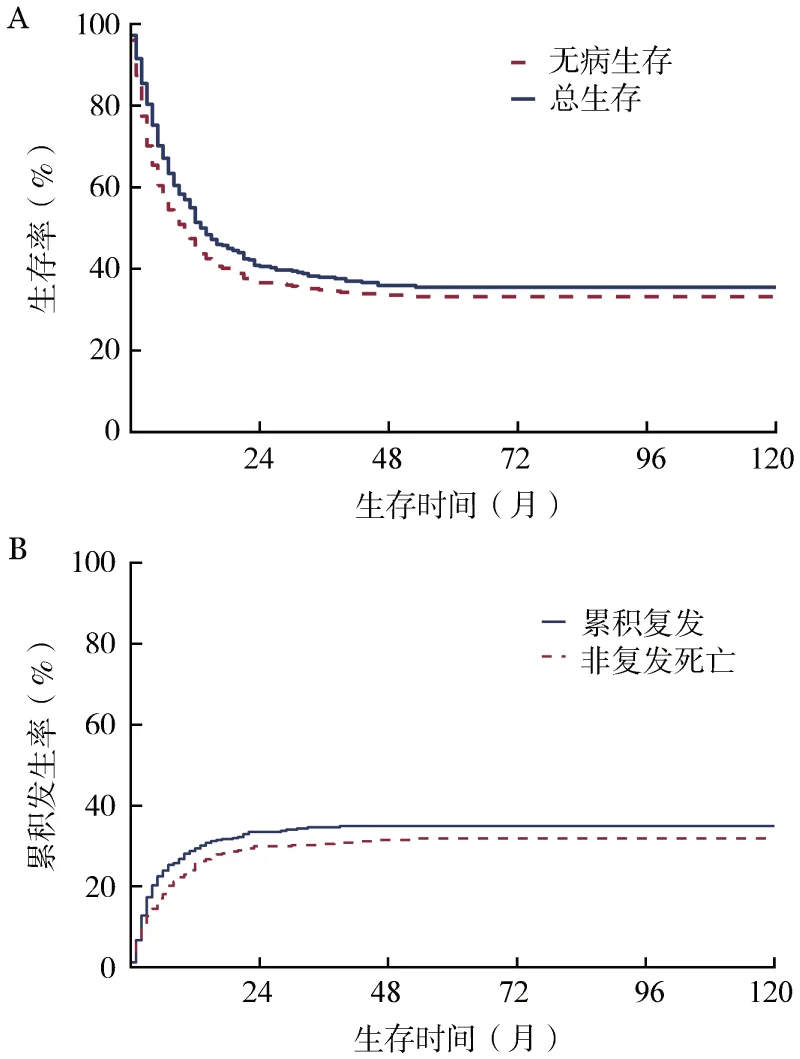
接受异基因造血干细胞移植治疗的510例活动性复发难治性急性髓系白血病患者的生存（A）和复发（B）曲线

3. 死亡情况：中位随访时间50（0～151）个月，随访截止时，299例患者发生死亡，其中复发仍为主要死亡原因，占49.8％，其次28.4％死于感染（表2）。预估10年NRM为31.9％（95％*CI*：27.6％～36.1％）（图1）。

**表2:** 接受异基因造血干细胞移植治疗的510例活动性复发难治性急性髓系白血病患者的死亡原因分析

死亡原因	例数（％）
复发	149（49.8）
感染	85（28.4）
慢性移植物抗宿主病	30（10.0）
急性移植物抗宿主病	16（5.4）
血栓性微血管病	10（3.3）
弥漫性肺泡出血	4（1.3）
脑卒中	2（0.7）
肝静脉闭塞病	1（0.3）
心源性猝死	2（0.7）

4. 预后因素分析：单因素分析纳入的变量包括供患者性别及年龄、ELN风险分层、骨髓原始细胞比例、髓外病灶、移植类型、CMV再激活、GVHD发生情况、发病至移植时间间隔、MNC和CD34^+^细胞输注量；将单因素分析中*P*<0.10的变量（附表1，**扫描本文首页二维码查阅**）以及临床上已知与预后相关的重要因素纳入Cox回归模型进行多因素分析（表3），结果显示，ELN风险分层是影响预后的关键因素，与低危组相比，中危及高危组的死亡风险分别增加了74％（*HR*＝1.74，*P*＝0.002）和97％（*HR*＝1.97，*P*<0.001）。同时发现发病至移植时间间隔≤10个月（*HR*＝0.64，*P*<0.001）是影响OS的独立保护因素。而CD34^+^细胞输注量<4.3×10⁶/kg的患者显出更差的生存（*HR*＝1.57，*P*<0.001）。值得注意的是，在校正混杂因素后，移植类型（单倍体对其他供者）对OS和DFS的影响均无统计学意义（均*P*>0.05）。

**表3:** allo-HSCT治疗的活动性复发难治性急性髓系白血病患者预后多因素分析

变量	总生存		无病生存		累积复发		非复发死亡	
	*HR*（95％ *CI*）	*P*值	*HR*（95％ *CI*）	*P*值	*HR*（95％ *CI*）	*P*值	*HR*（95％ *CI*）	*P*值
女性	1.00（0.79～1.27）	0.985	1.08（0.86～1.36）	0.488	1.20（0.88～1.63）	0.250	0.96（0.68-1.36）	0.811
ELN危险度	低危为参照		低危为参照		低危为参照		低危为参照	
中危	1.74（1.23～2.47）	0.002	1.77（1.27～2.48）	<0.001	2.73（1.61～4.64）	<0.001	0.98（0.64～1.51）	0.934
高危	1.97（1.38～2.81）	<0.001	1.99（1.41～2.80）	<0.001	2.90（1.69～4.95）	<0.001	0.88（0.55～1.39）	0.578
骨髓原始细胞比例	5％～<20％为参照		5％～<20％为参照		5％～<20％为参照		5％～<20％为参照	
20％～<50％	0.98（0.69～1.38）	0.900	1.07（0.77～1.48）	0.700	1.18（0.73～1.88）	0.499	0.95（0.60～1.50）	0.825
≥50％	1.18（0.86～1.62）	0.293	1.29（0.95～1.75）	0.109	1.62（1.03～2.55）	0.037	0.90（0.59～1.37）	0.617
CNSL	1.08（0.69～1.69）	0.731	1.16（0.76～1.78）	0.492	1.01（0.55～1.86）	0.980	1.41（0.83～2.40）	0.207
髓外病灶（除外CNSL）	1.03（0.72～1.46）	0.877	0.96（0.68～1.35）	0.825	0.91（0.56～1.47）	0.707	1.12（0.70～1.78）	0.648
移植类型	单倍体为参照		单倍体为参照		单倍体为参照		单倍体为参照	
同胞全合	0.83（0.58～1.19）	0.313	0.86（0.60～1.21）	0.386	1.09（0.71～1.69）	0.686	0.76（0.45～1.31）	0.330
非血缘	0.63（0.37～1.08）	0.092	0.69（0.42～1.13）	0.142	0.76（0.36～1.60）	0.469	0.78（0.37～1.60）	0.493
脐血	0.63（0.28～1.41）	0.260	0.72（0.34～1.53）	0.395	0.29（0.06～1.35）	0.113	1.52（0.67～3.44）	0.318
供者为女性	0.85（0.64～1.14）	0.286	0.83（0.62～1.10）	0.191	1.03（0.72～1.48）	0.871	0.68（0.44-1.04）	0.075
CMV再激活	1.07（0.84～1.37）	0.559	0.94（0.74～1.18）	0.573	1.00（0.73～1.37）	0.992	0.97（0.69-1.37）	0.865
aGVHD								
Ⅰ～Ⅱ级	1.17（0.90～1.54）	0.241	1.15（0.89～1.49）	0.295	0.99（0.71～1.39）	0.969	1.25（0.85-1.84）	0.258
Ⅲ～Ⅳ级	1.65（1.17～2.33）	0.004	1.40（1.00～1.96）	0.050	0.54（0.31～0.95）	0.033	2.36（1.50-3.72）	<0.001
患者年龄≤35岁	0.79（0.61～1.02）	0.075	0.87（0.68～1.10）	0.247	1.28（0.91～1.78）	0.152	0.68（0.47～0.97）	0.033
发病至移植时间间隔≤10个月	0.64（0.50～0.81）	<0.001	0.62（0.49～0.79）	<0.001	0.81（0.59～1.12）	0.198	0.74（0.53～1.03）	0.073
供者年龄≤35岁	1.14（0.88～1.48）	0.306	1.12（0.87～1.43）	0.375	0.91（0.66～1.24）	0.546	1.28（0.86～1.90）	0.227
MNC输注量<9.9×10^8^/kg	1.03（0.81～1.33）	0.794	0.99（0.77～1.25）	0.903	0.97（0.69～1.36）	0.859	1.08（0.76～1.54）	0.668
CD34^+^细胞输注量<4.3×10^6^/kg	1.57（1.22～2.03）	<0.001	1.62（1.27～2.08）	<0.001	1.64（1.18～2.28）	0.003	1.08（0.76～1.53）	0.670

**注** allo-HSCT：异基因造血干细胞移植；ELN：欧洲白血病网；CNSL：中枢神经系统白血病；CMV：巨细胞病毒；aGVHD：急性移植物抗宿主病；cGVHD：慢性移植物抗宿主病；MNC：单个核细胞

复发风险方面，多因素Fine-Gray模型显示，ELN高危分层（*HR*＝2.90，*P*<0.001）及移植前骨髓原始细胞≥50％（*HR*＝1.62，*P*＝0.037）与较高的CIR独立相关。此外，CD34⁺细胞输注量不足显著增加复发风险（*HR*＝1.64，*P*＝0.003）。Ⅲ～Ⅳ级aGVHD（*HR*＝0.54，*P*＝0.033）与低复发风险相关。

针对NRM的分析显示，Ⅲ～Ⅳ级aGVHD（*HR*＝2.36，*P*<0.001）是导致非复发死亡的首要原因。年龄≤35岁患者的非复发死亡风险较低（*HR*＝0.68，*P*＝0.033）。

## 讨论

R/R AML患者的OS率较低，预后不良，尤其是存在活动性疾病的患者，生存结局进一步恶化[12]–[14]。当前针对R/R AML的基础挽救手段仍为诱导缓解后桥接移植，但患者常伴随严重的感染和器官功能不全，无法顺利过渡至移植阶段，最终影响预后，因此，对于移植前仍存在活动性疾病的R/R AML患者，是否可以直接行挽救性移植成为关注的重点。本研究观察本中心移植前NR状态的患者行挽救性移植的疗效，10年OS率高于既往部分研究报道，本队列的随访时间明显长于多数既往研究[15]–[18]，为长期生存结局的评估提供了更为充分的依据。

本研究发现，ELN风险分层在OS、DFS及CIR中具有稳定的独立预测作用，ELN风险升高可增加患者的CIR，进而进一步降低OS率和DFS率，另外该分层对NRM无显著影响，提示疾病的分子生物学特征在决定患者的复发和长期生存起着关键作用。而肿瘤负荷仅被发现在CIR中作为独立的危险因素，未对OS、DFS、NRM有明显影响。这一结果表明，肿瘤负荷主要通过影响疾病复发过程而发挥作用，而对长期生存的影响受到其他因素的调节，从机制上表明，较高的肿瘤负荷通常代表更强的肿瘤细胞增殖活性和更大的残留白血病细胞群体，从而增加移植后复发的风险，当结合ELN风险分层即疾病本身的分子遗传学特征后，其对OS的影响降低，表明在活动性R/R AML患者，疾病本身的生物学特征相较于肿瘤负荷本身对长期预后更可能发挥决定性作用[19]，这一结果与近期ASAP研究结果相一致[20]。因此，在部分患者中，单纯追求移植前CR状态可能并非改善长期预后的唯一关键策略。

在移植时机方面，本研究发现发病至移植时间间隔较短（≤10个月）与更好的OS和DFS有关，提示此类患者尽早接受移植可能会通过减少疾病的进展及反复治疗而带来的相关损伤，从而改善整体的结局。CD34^+^细胞的输注量较高亦与更好的OS和DFS及较低的CIR有关，而对NRM无显著的影响，提示其主要通过促进造血及免疫重建，增强GVL效应降低复发风险，从而改善预后。既往研究亦提示CD34^+^细胞剂量与预后之间可能存在非线性关系，因此需进一步明确最佳输注量范围[21]–[22]。

在非复发死亡方面，本研究除了发现Ⅲ～Ⅳ级aGVHD和重度cGVHD的发生是非复发死亡的主要危险因素，还发现了患者移植时年龄较大亦与NRM增加有关，但并未影响患者的总体预后，进一步表明年龄不再是移植的绝对禁忌证，应结合总体情况进行综合评估。

综上所述，本研究基于大样本真实世界数据表明，在移植前仍存在活动性疾病的R/R AML患者中，挽救性allo-HSCT仍可为部分患者带来长期生存获益。预后受多重因素共同影响，ELN风险分层及cGVHD是复发的重要影响因素，而非复发死亡则主要与aGVHD、重度cGVHD及患者年龄相关。相比之下，移植前肿瘤负荷虽影响复发风险，但并非决定长期生存的核心因素。上述结果提示，在临床实践中，应更加重视生物学风险分层、移植时机选择及移植物免疫效应的调控，而非单纯追求移植前完全缓解状态。

尽管本研究基于大样本真实世界研究且具有较长的随访时间，但仍是一项单中心回顾性研究，存在残余混杂偏倚及选择偏倚。同时本研究实践跨度较长，不同时期的移植策略、移植后维持治疗及感染防治水平的改善会对患者预后产生影响。此类患者生物学特征复杂，既往治疗方案及疾病状态存在显著异质性，因此结果需要进一步通过前瞻性及多中心研究进行验证。

## Supplementary Material


